# Does Off-Pump Coronary Artery Bypass Grafting Negatively Impact Long-Term Survival and Freedom from Reintervention?

**DOI:** 10.1155/2013/602871

**Published:** 2013-09-11

**Authors:** Shahzad G. Raja, Mubassher Husain, Florentina L. Popescu, Dimple Chudasama, Siobhan Daley, Mohamed Amrani

**Affiliations:** ^1^Department of Cardiac Surgery, Harefield Hospital, Hill End Road, Harefield, London UB9 6JH, UK; ^2^Department of Quality & Safety, Harefield Hospital, Hill End Road, Harefield, London UB9 6JH, UK

## Abstract

Recently published evidence has raised concerns about worse late mortality and increasing need for reintervention after off-pump coronary artery bypass grafting. We undertook this study to assess the impact of off-pump coronary artery bypass grafting on survival and freedom from reintervention at 10 years. From January 2002 to December 2002, 307 consecutive patients who had isolated multivessel off-pump coronary artery bypass grafting at our institution were compared to a control group of 397 patients that underwent multivessel on-pump coronary artery bypass grafting during the same period. In addition, univariate and risk-adjusted comparisons between the two groups were performed at 10 years. Kaplan-Meier survival was similar for the two cohorts. After adjusting for clinical covariates, off-pump coronary artery bypass grafting did not emerge as a significant independent predictor of long-term mortality (Hazard Ratio 0.91; 95% Confidence Interval 0.70–1.12), readmission to hospital for cardiac cause (Hazard Ratio 0.96; 95% Confidence Interval 0.78–1.10), or the need for reintervention (Hazard Ratio 0.93; 95% Confidence Interval 0.87–1.05). Off-pump coronary artery bypass grafting compared with on-pump coronary artery bypass grafting does not adversely impact survival or freedom from reintervention at a 10-year follow-up.

## 1. Introduction

For decades cardiac surgeons have been used to performing delicate coronary anastomoses on cardiopulmonary bypass (CPB). However, the price of a still and bloodless field is ultimately paid by the patients in the form of sequelae of negative effects of CPB including blood trauma, activation of a series of inflammatory responses, nonpulsatile flow, and possible embolization of air or debris—most particularly embolization of atherosclerotic debris from the aorta. Off-pump coronary artery bypass (OPCAB) grafting was rediscovered with the primary objective of avoiding these deleterious effects of CPB [1]. Most published studies comparing these two techniques of coronary artery bypass grafting have shown that results of OPCAB are comparable to those of on-pump grafting [2–7]. Evidence in the form of randomized controlled trials and observational studies as well as meta-analyses has demonstrated decreased length of hospitalization, myocardial enzyme release, incidence of atrial fibrillation, and blood product utilization with OPCAB grafting [2–7]. To all these advantages we can add the benefits of shorter respiratory support, and fewer cases of pulmonary dysfunction and abnormal renal function [8–11]. Despite increasing recognition of the benefits of OPCAB grafting, concerns persist regarding its impact on long-term mortality and freedom from reintervention [12–15]. 

We undertook this study to assess the impact of OPCAB grafting on long-term outcomes.

## 2. Methods

### 2.1. Study Sample

This study comprised a retrospective analysis of a prospectively collected cardiac surgery database (PATS; Dendrite Clinical Systems, Ltd, Oxford, UK) as well as a follow-up questionnaire approved by the institutional ethics committee. Due to its retrospective nature informed consent was waived for this study. The PATS database captures detailed information on a wide range of preoperative, intraoperative, and hospital postoperative variables (including complications and mortality) for all patients undergoing cardiac surgery in our institution. The database was collected and reported in accordance with the Society for Cardiothoracic Surgery in Great Britain & Ireland database criteria. In addition, the medical notes and charts of all the study patients were reviewed. For information on long-term outcomes, a questionnaire was mailed to all surviving patients or to the general practitioners of those patients who had died during the follow-up period.

From January 2002 to December 2002, 307 consecutive patients that underwent isolated multivessel OPCAB grafting at our institution were compared to a control group of 397 patients that underwent multivessel on-pump coronary artery bypass grafting during the same period. Patient characteristics of both groups are shown in Table 1. This particular patient cohort was selected for two reasons. Firstly, to have a follow-up that is truly long termed and secondly to exclude the influence of learning curve which is a well-recognised influence on outcomes [16]. The surgeons contributing OPCAB patients to this study had on an average performed ≥100 OPCAB procedures individually since the inception of the OPCAB programme at our institution in late 1996 and hence were assumed to have traversed their learning curve. Indications for surgical intervention were determined at a weekly review involving cardiologists, cardiac surgeons, and cardiac radiologists. Patients were placed on a specific waiting list according to the urgency of their procedure. 

### 2.2. Operative Technique

Four surgeons performed both on-pump and OPCAB operations during the study period. All interventions were performed via a midline sternotomy. The choice of on- or off-pump strategy was based on surgeon's preference. Left and right internal mammary arteries (IMAs) were harvested with minimal trauma as pedicled or skeletonized grafts, based on surgeon's preference, and treated with papaverine solution prior to use. Great saphenous vein was harvested using open technique.

Conventional coronary artery bypass grafting on CPB was performed at 34°C. CPB was instituted with single two-stage right atrial cannulation and an ascending aorta perfusion cannula. Standard bypass management included membrane oxygenators, arterial line filters, and nonpulsatile flow of 2.4 L/min/m^2^, with a mean arterial pressure greater than 50 mm Hg. The myocardium was protected by using intermittent antegrade cold blood cardioplegia (4 : 1 blood to crystalloid ratio). Anticoagulation was achieved using 300 U/kg of heparin. If required, heparin was supplemented to maintain the activated clotting time above 480 seconds and was reversed by protamine at the end of the procedure.

All patients underwent conventional multivessel CABG using varying combinations of left and/or right IMA and saphenous vein grafts. All distal and proximal anastomoses on CPB were performed during a period of single aortic cross-clamping. 

For off-pump CABG the heart was stabilized using the suction-irrigation tissue stabilization system. A deep pericardial retraction suture helped position the heart for grafting. Anticoagulation was achieved with 150 U/kg of heparin. If required, heparin was supplemented to maintain the activated clotting time above 250 seconds and was reversed by protamine at the end of the procedure. Blood pressure was continually optimized during the procedure, and the mean arterial pressure was maintained above 50 mm Hg by repositioning the heart and by intravenous fluids or selective use of vasoconstrictors, or both. The proximal graft anastomoses to the aorta were performed with partial cross-clamping of the ascending aorta. Each distal anastomosis was followed by construction of the corresponding proximal anastomosis.

### 2.3. Postoperative Management

Postoperative intensive care unit management was standardized for all patients. All patients received intravenous nitroglycerin (0.1 to 8 *μ*g· kg^−1^·min^−1^) infusions for the first 24 hours unless hypotensive (systolic blood pressure < 90 mm Hg). Choice of inotropic agents was dictated by the hemodynamic data. Other routine medications included daily aspirin and resumption of cholesterol-lowering agents and *β*-blockers. Diuretics, angiotensin-converting enzyme inhibitors, and warfarin were gradually introduced when indicated clinically.

### 2.4. Variables and Data Collection

Preoperative variables of interest included age, sex, body mass index (BMI), smoking history, chronic obstructive pulmonary disease, diabetes, hypercholesterolemia, renal insufficiency (preoperative serum creatinine ≥ 200 *μ*mol·L^−1^), hypertension, peripheral vascular disease, cerebrovascular disease, left ventricular ejection fraction, urgency (operation performed < 24 h versus > 24 h from time of referral), previous myocardial infarction (MI), prior percutaneous coronary interventions (PCI), preoperative intravenous nitrates, preoperative intravenous inotropes, number of diseased vessels, preoperative intra-aortic balloon pump (IABP), and logistic EuroSCORE. Intraoperative variables of interest included types of grafts used, grafts/patient, cardiopulmonary bypass (CPB) time, aortic cross-clamp time, conversion to CPB, and index of completeness of revascularization (ICOR). The ICOR was defined as the total number of distal grafts constructed divided by the number of the affected coronary vessels reported on the preoperative coronary angiogram [17]. Complete revascularization was assumed when the ICOR was ≥1.

Postoperative variables of interest included in-hospital mortality, postoperative IABP, stroke or transient ischemic attack (TIA), prolonged ventilation > 24 hours, atrial fibrillation, deep sternal infection, superficial sternal infection, mediastinitis, vein harvest site infection, blood products usage, hemofiltration, inotropes leaving operating room (OR), chest infection, return to OR for bleeding, gastrointestinal complications, and length of intensive care unit (ICU) and hospital stay.

The long-term outcomes of interest were all-cause mortality following discharge from hospital, coronary reintervention (percutaneous or coronary artery bypass grafting), or readmission for any cardiac cause defined by the following codes from the 9th revision of the International Classification of Disease, Clinical Modification [18]: 410 (acute MI), 411 (unstable angina), 412 (old MI), 413 (angina pectoris), 414 (other forms of chronic ischemic heart disease), 426 (conduction disorders), 427 (cardiac dysrhythmias), 428 (heart failure), 429 (ill-defined descriptions and complications of heart disease). 

### 2.5. Statistical Analysis

Patients, who underwent OPCAB grafting, were compared to those who did not, using *t*-tests and Kruskal-Wallis test for continuous variables and *χ* ^2^ test for categorical variables. A propensity analysis was performed modeling the probability of receiving OPCAB grafting. Briefly, a nonparsimonious multivariate logistic regression model using clinically relevant variables was generated to compute a propensity score for each patient. All clinically relevant variables were included in the model. The propensity score (or probability of receiving OPCAB grafting) was then used to obtain a one-to-one match of all OPCAB grafting cases with CPB controls by a “greedy  5 → 1  matching” technique [19]. In-hospital outcomes were compared between these matched groups. 

Logistic regression was used to examine the association of OPCAB grafting with in-hospital adverse events after adjusting for differences between patients on the basis of each of the above-mentioned preoperative variables. The association between OPCAB grafting and the long-term outcomes of interest was analyzed using adjusted survival curves and Cox proportional hazards modeling techniques. All baseline characteristics were included in the fully adjusted multivariate Cox models. 

Statistical significance was indicated by a two-tailed *P* value < 0.05. All analyses were performed with the Statistical Analysis Systems software package (Release 9.1.3; SAS Institute, Cary, NC). The authors had full access to the data and take responsibility for its integrity. All authors have read and agree to the paper as written.

## 3. Results

A total of 704 patients formed the final study population. Compared to patients who had on-pump grafting, those receiving OPCAB grafting were more likely to be male and more likely to have diabetes, hypercholesterolemia, renal insufficiency, peripheral vascular disease, two-vessel disease, and elective surgery (Table 1). Off-pump grafting patients also received more bilateral IMAs than control group (39.7% versus 15.4%; *P* < 0.01) as listed in Table 2. Overall, there were fewer distal anastomoses performed in OPCAB group compared to control (2.91 ± 1.06 grafts versus 3.4 ± 0.4 grafts; *P* < 0.01). Unadjusted hospital mortality was 1.3% for OPCAB group and 1.5% for control group (*P* = 0.76). The overall in-hospital mortality for the entire cohort was 1.4%.

The propensity score model included 26 patient variables listed in Table 1. The *c* statistic for this model was 0.81 (Hosmer-Lemeshow goodness-of-fit *P* = 0.3057). All 307 OPCAB grafting cases could be matched to 307 control patients. The two groups were well matched for all the patient variables (Table 3).

The in-hospital mortality for the propensity-matched OPCAB group was similar to the control group (1.3% versus 1.6%; *P* = 0.71). The length of hospitalization was a median of 7 days in both groups with an interquartile (IQR) range of 4 to 13 days (*P* = 0.98). Major morbidity was not statistically different between OPCAB and matched groups (Table 4). However, significantly more patients in the control group required inotropes (17.6% versus 8.5%; *P* < 0.001), hemofiltration (6.2% versus 1.3%; *P* = 0.01), received blood products (29.6% versus 6.2%; *P* < 0.001), and were reexplored for bleeding (5.5% versus 2.6%; *P* = 0.01) compared with matched OPCAB patients. After adjusting for clinical covariates, OPCAB grafting was not an independent predictor of in-hospital adverse events (odds ratio (OR) 0.78, 95% confidence interval (CI) 0.66–0.85, *P* = 0.31).

The follow-up was 100% complete at 10 years. Over the entire follow-up period, 11 (3.6%) patients died in the OPCAB group and 19 (4.8%) in the control group (*P* = 0.67). After adjusting for clinical covariates, OPCAB grafting did not emerge as a significant independent predictor of long-term mortality: the hazard ratio (HR) was 0.91 (95% CI 0.70–1.12, *P* = 0.87). Risk-adjusted survival was 85% after OPCAB grafting and 84% after on-pump grafting (*P* = 0.89) during the long-term follow-up. After discharge, 3.3% of OPCAB grafting patients and 3.8% of on-pump grafting patients were readmitted to hospital for cardiac reasons (*P* = 0.81). These included 2 (0.7%) OPCAB grafting and 3 (0.9%) on-pump grafting patients who were readmitted for repeat revascularization (percutaneous or surgical; *P* = 0.93); repeat coronary artery bypass grafting was performed in 1 (0.3%) OPCAB and 1 (0.3%) on-pump grafting patient (*P* = 1.00). After adjusting for clinical covariates, OPCAB grafting did not emerge as a significant independent predictor of readmission to hospital for cardiac cause (HR 0.96; 95% CI 0.78–1.10) or the need for reintervention (HR 0.93; 95% CI 0.87–1.05). 

## 4. Discussion

The results of our study comparable to those previously reported by Puskas et al. [20] and Angelini et al. [21] confirm that OPCAB grafting is associated with similar in-hospital and long-term outcomes compared with on-pump grafting. There is a large body of scientific evidence validating benefits of OPCAB grafting for myocardial revascularization. These benefits include improved in-hospital as well as mid-term outcomes [4–11]. However, concerns persist regarding its impact on long-term mortality and freedom from reintervention [12–15].

Takagi and associates have recently published a meta-analysis of randomized controlled trials suggesting that OPCAB grafting may increase late (≥1 year) all-cause mortality by a factor of 1.37 over on-pump grafting [14]. However, the findings of this meta-analysis must be interpreted with caution as the results are strongly influenced by the ROOBY trial [22] which has attracted a lot of criticism and has several important limitations. It is a well-established fact that incomplete revascularization and lower graft patency have a negative impact on long-term survival. Bell and colleagues performed a retrospective analysis of 3,372 nonrandomized surgical patients from the Coronary Artery Surgery Study Registry (3-vessel coronary disease) [23]. In patients having class I or II angina (Canadian Cardiovascular Society criteria), adjusted cumulative 4-year survivals according to the number of vessels bypassed were 85% (1 vessel), 94% (2 vessels), 96% (3 vessels), and 96% (more than 3 vessels) (*P* = 0.022). Placing grafts to 3 or more vessels was independently associated with improved survival (RR, 0.745; 95% CI, 0.591 to 0.940; *P* = 0.0132) in patients having class III or IV angina. Some of the major criticisms of OPCAB grafting have been low revascularization rates and suboptimal anastomotic quality resulting in poor graft patency and long-term outcomes [12–15]. These concerns are no longer valid particularly in large-volume centres and for surgeons who have traversed their learning curve. Our results strongly back this claim as we have clearly shown that all patients in our study had complete revascularization (ICOR ≥ 1) translating into improved long-term outcomes. Similar findings have been reported by Puskas and associates for their SMART trial [3, 20]. 

We have attempted to make meaningful comparisons between the OPCAB grafting group and a contemporaneous group of on-pump grafting control patients. To do this we have used two statistical approaches based on propensity modeling, a technique that has been strongly advocated in several recent publications, in an effort to better evaluate treatment comparisons from nonrandomized clinical experiences [24]. The propensity score is the probability of a patient receiving a given intervention (in this case OPCAB grafting) based on a nonparsimonious model derived from preoperative patient variables. The propensity model thus reduces many variables to a single balancing score, facilitating meaningful intergroup comparisons. We used two approaches, namely, the creation of matched pairs based on propensity score and logistic regression analysis of outcomes in which propensity score participated as a variable. 

Using the propensity matching technique, the OPCAB and control groups were remarkably well matched in terms of known risk predictors of outcomes after coronary artery bypass grafting. The overall mortality and major morbidity between groups were not statistically different. However, the incidence of reexploration for bleeding and transfusion of blood products in the on-pump group was significantly higher than that of the OPCAB group. The (2.2% versus 5.5%) incidence of reexploration for bleeding in this study compares quite well with incidences of 2% to 6% mentioned in the literature [25]. Continuation of aspirin until the day of surgery and increased number of distal anastomoses with an increased number of potential bleeding sites in the on-pump patients could be some of the plausible explanations for this phenomenon. Additionally, it is well established that patients undergoing OPCAB do not show any impairing effect of CPB on hemostasis [26]. Because of the absence of the artificial surfaces of the heart-lung machine, the various platelet activation mechanisms and depletion caused by contact activation with extracorporeal surfaces, bubble oxygenator, cardiotomy suction, and filters are avoided leading to reduced postoperative bleeding [26]. In addition, excessive bleeding may be related to a coagulopathy resulting from greater heparin doses during CPB as guided by dosing protocols based on body weight and activated coagulation time (ACT) values or with maintenance of a defined heparin concentration [26]. In contrast, a low level of intraoperative heparinization in OPCAB patients preserves hemostasis [26]. Finally, markedly reduced systemic inflammatory response after OPCAB surgery may also contribute to reduction in postoperative blood loss [27].

Additional advantage of OPCAB grafting was significantly less need for hemofiltration despite the significantly more patients having preoperative serum creatinine ≥200 *μ*mol·L^−1^. Patients undergoing coronary artery bypass grafting have several risk factors that predispose them to develop acute kidney injury (AKI). These include but are not limited to advanced age and presence of multiple comorbid illnesses like diabetes, hypertension, congestive heart failure, peripheral vascular disease, and most importantly preexisting renal insufficiency [28]. However, the risk of developing AKI is related to the surgical procedure itself. Some of the major causes are the application of CPB circuit that requires placement of aortic cross-clamp and the inevitable reduction in blood supply, albeit for a short time, and loss of pulsatile blood flow to the kidney [28]. In addition, exposure of blood to circuit membranes stimulates release of inflammatory mediators like catecholamines and free hemoglobin that may be involved in the development of AKI [28]. The length of the use of the bypass circuit further dictates likelihood of development of AKI. At a pathological level, although no biopsy-based studies have been done, based on the pathophysiology of developing AKI, acute tubular necrosis is suspected to be the most likely cause [28]. There is evidence both from randomized controlled trials as well as observational studies that avoiding CPB may reduce the AKI risk as OPCAB grafting is not associated with constellation of changes described above [28].

Another important finding of this study was the increased use of bilateral IMAs in patients receiving OPCAB grafting. There is evidence to support the concept that the greater the number of arterial conduits used, the better are the long-term results [29]. Two meta-analyses have proven the advantages of bilateral IMA grafting compared with single IMA grafting [30, 31]. As more patients in the OPCAB cohort had two-vessel coronary artery disease they were possibly preferentially offered two IMAs in order to achieve complete revascularization. This revascularization strategy not only offered a survival benefit but also reduced the need for reintervention. 

**Figure 1:**
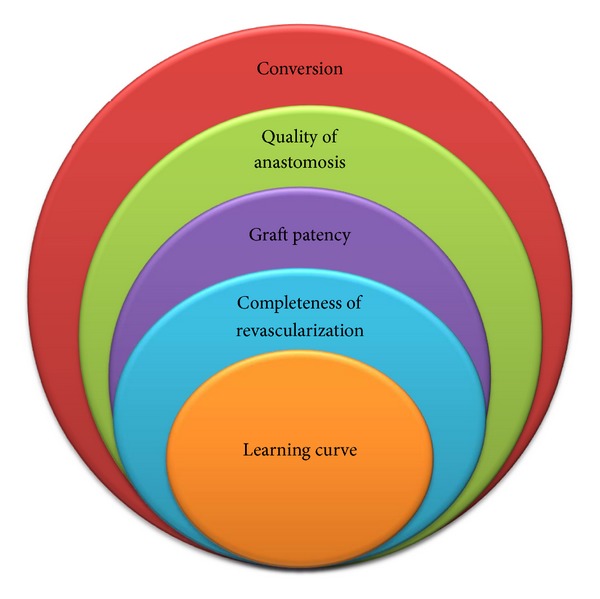
Concerns associated with off-pump coronary artery bypass grafting.

Finally, it is extremely important to highlight that central to all the concerns associated with OPCAB grafting is the issue of learning curve (Figure 1). Surgeons of low or even moderate OPCAB experience have been found to be predictive of emergency conversion [32] as well as responsible for poor graft patency and incomplete revascularization [33]. The technical difficulty of OPCAB grafting means it involves a steep learning curve that applies to both trainees and consultant surgeons new to OPCAB grafting. The key skill in OPCAB surgery is to be able to perform coronary anastomoses on a beating target myocardium rather than a stationary one. Exposure to OPCAB techniques during training is infrequent and the acquisition of proficiency even less so. In a study of residents undergoing cardiothoracic training in the United States, only 22% of residents had performed 20 or more OPCAB procedures during their training [34]. Of these, only 4% had performed OPCAB circumflex coronary artery revascularization. Similarly in the United Kingdom, only 51% of trainees surveyed (76% of all trainees) had experienced OPCAB in their training program, although 96% believed that OPCAB training was essential [35]. Among established surgeons, the adoption of OPCAB has also been highly variable with rates varying between zero and 100% of revascularization cases per surgeon, even within a single institution. The reasons for the variation in the adoption of OPCAB techniques are multifactorial. They include the lack of established training programs, the perception that success with the technique is limited to more proficient surgeons, and a fear of deleterious patient outcomes, especially during the learning curve [35].

The learning curve in OPCAB surgery can be safely negotiated with appropriate patient selection, individualized grafting strategy, peer-to-peer training of the entire team, and graded clinical experience (preoperative planning, adequate exposure, proximal anastomoses to the aorta, and distal anastomoses initially to anterior wall vessels, followed by inferior wall vessels and then lateral wall vessels) [36]. In our experience, the surgeon's learning curve is around 75 to 100 cases, and good proficiency with the technique is usually associated with a low 1% to 2% conversion rate and good short- as well as long-term outcomes as shown by the findings of this study. 

The primary limitation of the study is its retrospective nature. Propensity score adjustment is no substitute for a properly designed, randomized controlled trial. The retrospective nature of the study cannot account for the unknown variables affecting the outcome that are not correlated strongly with measured variables. However, retrospective comparisons with propensity score adjustment are more versatile and offer a useful way of interpreting large amounts of audit data and of seeking answers to questions that may present insuperable difficulties in the design of randomized controlled trials. Despite the retrospective and observational nature of the study, we provided data on a large cohort of patients undergoing OPCAB grafting for comparison with on-pump grafting control group, with the longest follow-up which has not been reported before, and demonstrated the safety of OPCAB grafting as well as its potential for providing complete revascularization that translates into long-term outcomes comparable with a contemporaneous cohort of on-pump grafting patients that also underwent complete revascularization. Lastly, our analysis would have been substantially enhanced if long-term graft patency comparisons were available. However, due to costs, routine follow-up coronary angiography was not performed. The need for coronary angiography was dictated by the occurrence of angina, instability, or electrocardiogram changes in the perioperative or late follow-up period. 

In summary, OPCAB grafting compared with on-pump coronary artery bypass grafting does not adversely impact survival or freedom from reintervention at 10-year follow-up. 

## Figures and Tables

**Table 1:** Unmatched preoperative patient characteristics.

Variable	Off-pump (*n* = 307)	On-pump (*n* = 397)	*P* value
Age (years ± SD)	62.3 ± 11.8	62.7 ± 9.9	0.91
Male	219 (71.3)	216 (54.4)	0.02
BMI	29.1 ± 4.0	28.7 ± 4.1	0.87
Diabetes	108 (35.2)	106 (26.7)	0.03
Hypertension	163 (53.1)	212 (53.4)	0.97
Never smoked	99 (32.2)	137 (34.5)	0.76
Hypercholesterolemia	139 (45.3)	157 (39.5)	0.04
COPD	25 (8.1)	33 (8.3)	0.91
CCS ≥ 3	79 (25.7)	101 (25.4)	0.97
NYHA ≥ 2	166 (54.1)	219 (55.2)	0.87
PVD	27 (8.8)	21 (5.3)	0.03
MI in 30 days prior to CABG	79 (25.7)	101 (25.4)	0.99
Preoperative serum creatinine ≥ 200 *μ*mol·L^−1^	13 (4.2)	7 (1.8)	0.04
<30% ejection fraction	16 (5.2)	22 (5.5)	0.93
30–49% ejection fraction	69 (22.5)	98 (24.7)	0.87
≥50% ejection fraction	222 (72.3)	277 (69.8)	0.76
Preoperative IV nitrates	20 (6.5)	31 (7.8)	0.65
Preoperative IV inotropes	1 (0.3)	2 (0.5)	0.79
Preoperative IABP	19 (6.2)	24 (6.0)	0.97
Previous PCI	13 (4.2)	17 (4.2)	1.0
CVA/TIA	5/4 (2.9)	3/9 (3.0)	0.91
LMS stenosis > 50%	119 (38.8)	159 (40.0)	0.6
Two vessels	112 (36.5)	110 (27.7)	0.04
Three vessels	195 (63.5)	287 (72.3)	0.06
Urgent	101 (32.9)	161 (40.6)	0.03
Logistic EuroSCORE (mean ± SD)	3.3 ± 3.4	3.4 ± 3.6	0.76

Values in parentheses are percentages.

BMI: body mass index; CABG: coronary artery bypass grafting; CCS: Canadian Cardiovascular Society; COPD: chronic obstructive pulmonary disease; CVA: cerebrovascular accident; IABP: intra-aortic balloon pump; LMS: left main stem; MI: myocardial infarction; NHYA: New York Heart Association; PCI: percutaneous coronary intervention; PVD: peripheral vascular disease; SD: standard deviation; TIA: transient ischemic attack.

**Table 2:** Unmatched intraoperative data.

Variable	Off-pump (*n* = 307)	On-pump (*n* = 397)	*P* value
LIMA usage	307 (100)	397 (100)	1.00
RIMA usage	122 (39.7)	61 (15.4)	<0.01
SVG usage	185 (60.3)	336 (84.6)	<0.01
Grafts/patient	2.91 ± 1.06	3.4 ± 0.4	<0.01
CPB time (min)	—	79.7 ± 35.2	—
Aortic cross-clamp time (min)	—	49.4 ± 29.5	
Conversion to CPB	3 (0.9)	—	—
ICOR	1.09 ± 0.17	1.11 ± 0.19	0.87

Values in parentheses are percentages.

CPB: cardiopulmonary bypass; ICOR: index of completeness of revascularization; LIMA: left internal mammary artery; RIMA: right internal mammary artery; SVG: saphenous vein graft.

**Table 3:** Preoperative characteristics of propensity-matched patients.

Variable	Off-pump (*n* = 307)	On-pump (*n* = 307)	*P* value
Age (years ± SD)	62.3 ± 11.8	62.6 ± 7.9	0.93
Male	219 (71.3)	211 (68.7)	0.87
BMI	29.1 ± 4.0	28.6 ± 3.5	0.91
Diabetes	108 (35.2)	99 (32.2)	0.76
Hypertension	163 (53.1)	172 (56.0)	0.87
Never smoked	99 (32.2)	114 (37.1)	0.78
Hypercholesterolemia	139 (45.3)	143 (46.6)	0.83
COPD	25 (8.1)	27 (8.8)	0.91
CCS ≥ 3	79 (25.7)	82 (26.7)	0.87
NYHA ≥ 2	166 (54.1)	179 (58.3)	0.74
PVD	27 (8.8)	20 (6.5)	0.42
MI in 30 days prior to CABG	79 (25.7)	81 (26.4)	0.89
Preoperative serum creatinine ≥ 200 *μ*mol·L^−1^	13 (4.2)	7 (2.3)	0.34
<30% ejection fraction	16 (5.2)	19 (6.2)	0.83
30–49% ejection fraction	69 (22.5)	76 (24.8)	0.87
≥50% ejection fraction	222 (72.3)	212 (69.0)	0.81
Preoperative IV nitrates	20 (6.5)	23 (7.5)	0.87
Preoperative IV inotropes	1 (0.3)	1 (0.3)	1.0
Preoperative IABP	19 (6.2)	20 (6.5)	0.91
Previous PCI	13 (4.2)	13 (4.2)	1.0
CVA/TIA	5/4 (2.9)	3/6 (2.9)	1.0
LMS stenosis > 50%	119 (38.8)	159 (40.4)	0.54
Two vessels	112 (36.5)	101 (32.9)	0.29
Three vessels	195 (63.5)	209 (68.1)	0.45
Urgent	101 (32.9)	113 (37.1)	0.53
Logistic EuroSCORE (mean ± SD)	3.3 ± 3.4	3.3 ± 2.9	0.71

Values in parentheses are percentages.

BMI: body mass index; CABG: coronary artery bypass grafting; CCS: Canadian Cardiovascular Society; COPD: chronic obstructive pulmonary disease; CVA: cerebrovascular accident; IABP: intra-aortic balloon pump; LMS: left main stem; MI: myocardial infarction; NHYA: New York Heart Association; PCI: percutaneous coronary intervention; PVD: peripheral vascular disease; SD: standard deviation; TIA: transient ischemic attack.

**Table 4:** Postoperative data of propensity-matched patients.

Variable	Off-pump (*n* = 307)	On-pump (*n* = 307)	*P* value
Inotropes leaving OR	26 (8.5)	54 (17.6)	<0.001
Stroke/TIA	1/1 (0.7)	2/1 (1.0)	0.87
Atrial fibrillation	37 (12.1)	47 (15.3)	0.73
Chest infection	14 (4.6)	19 (6.2)	0.76
Hemofiltration	4 (1.3)	19 (6.2)	0.01
Postoperative IABP	5 (1.6)	7 (2.3)	0.79
Ventilation > 24 hrs	7 (2.3)	11 (3.6)	0.67
Superficial sternal infection	3 (0.9)	4 (1.3)	0.84
Deep sternal infection	2 (0.7)	3 (0.9)	0.91
Mediastinitis	0 (0)	1 (0.3)	0.76
Vein harvest site infection	7 (2.3)	10 (3.3)	0.83
Blood product usage	19 (6.2)	91 (29.6)	<0.001
Return to OR for bleeding	8 (2.6)	17 (5.5)	0.01
Tracheostomy	1 (0.3)	3 (0.9)	0.67
GI complications	2 (0.6)	3 (0.9)	0.79
Length of ICU stay median (IQR)	1 (1–3)	1 (1–3)	0.98
Length of hospital stay median (IQR)	7 (4–13)	7 (4–13)	0.98
In-hospital mortality	4 (1.3)	4 (1.3)	1.00
Late mortality	11 (3.6)	12 (3.9)	0.91
Readmission	10 (3.3)	11 (3.6)	0.93
Reintervention	2 (0.7)	2 (0.7)	1.00

Values in parentheses are percentages.

GI: gastrointestinal; IABP: intra-aortic balloon pump; ICU: intensive care unit; IQR: interquartile range; OR: operating room; TIA: transient ischemic attack.
